# Silver-Russell syndrome: phenotype features and oral health status

**DOI:** 10.1186/s13023-025-03886-y

**Published:** 2025-07-19

**Authors:** Paula Piekoszewska-Ziętek, Aneta Witt-Porczyk, Krystyna Chrzanowska, Małgorzata Zadurska, Dorota Olczak Kowalczyk

**Affiliations:** 1 Department of Paediatric Dentistry, Medical University of Warsaw, St. Binieckiego 6, 02-097 Warsaw, Poland; 2 Private Dental Practice, Warsaw, Poland; 3 Department of Medical Genetics, Children’s Memorial Health Institute, St. Dzieci Polskich 20, 04-736 Warsaw, Poland; 4 Department of Orthodontics, Medical University of Warsaw, St. Binieckiego 6, 02-097 Warsaw, Poland

**Keywords:** Silver-Russell syndrome, Oral cavity, Caries, Malocclusion, Gingivitis

## Abstract

**Background:**

Silver-Russell Syndrome is a rare malformation syndrome with a variable clinical and genetic presentation. Its incidence is estimated at 1:70.000–1:100.000 births. Since the diagnosis of Silver-Russell syndrome is based primarily on the identification of clinical features, studies assessing the craniofacial/dental changes present in this group of patients are important. The aim of the study was to evaluate phenotype features and oral health status in patients with Silver-Russell syndrome.

**Results:**

In the extraoral examination, patients with SRS were found to have a triangular facial shape, facial asymmetry, low-set, protruding ears, narrow lips and downward-facing mouth angles. In intraoral examination, reduced tongue dimensions, cleft palate and gothic palate were observed. There were no statistically significant differences in Plaque Index values between the groups. Gingival Index values were significantly higher in the Silver-Russell syndrome. The prevalence of caries was also higher in the group of subjects with Silver-Russell syndrome.

**Conclusions:**

Patients with Silver-Russell syndrome present themselves with features that affect oral health. Prompt orthodontic and dental intervention in children with SRS can help normalize oral function and facial appearance.

## Introduction

Silver-Russell Syndrome (SRS) is a rare malformation syndrome with a variable clinical and genetic presentation. Its incidence is estimated at 1:70.000–1:100.000 births [1, 2]. An essential role in the pathogenesis of the disease is played by mechanisms of epigenetic inheritance [2]. Genetic testing confirms the clinical diagnosis in about 60% of affected individuals. Hypomethylation of imprinted control region 1 (ICR1) at 11p15.5 causes SRS in 35–50% of patients, while maternal uniparental disomy (mUPD7) causes SRS in 7–10% [3]. There are a small number of people with SRS who have duplications, deletions or translocations involving imprinting centers at 11p15.5 or duplications, deletions or translocations involving chromosome 7. However, about 40% of those who meet clinical criteria for SRS have negative molecular and/or cytogenetic findings [3].

Patients with SRS exhibit pre- and postnatal growth retardation. Characteristics include low birth weight, poor postnatal growth and weight gain, short stature, characteristic dysmorphia and asymmetry, which may involve the face, limbs and/or trunk [4]. The phenotype in patients diagnosed with SRS syndrome changes with age. The most severe phenotype is observed in childhood. Patients exhibit a small, triangular face with a narrow chin and prominent frontal cusps, which may suggest macrocephaly. [5]. Within the skeletal system, congenital asymmetry of limb length, scoliosis, congenital hip dysplasia and clinodactyly of the hand’s fifth finger are observed. There may be “cafe au lait” type spots on the skin. Defects of the genitourinary system (for example, horseshoe kidney), as well as gastrointestinal disorders (including gastroesophageal reflux, food aversion or feeding problems), endocrine disorders (such as growth hormone deficiency and precocious puberty), are also frequently observed [2, 4]. A clinical diagnosis can be established in an individual who meets at least four NH-CSS (The Netchine-Harbison Clinical Scoring System) clinical criteria—prominent forehead/forehead convexity and relative macrocephaly at birth, plus two additional findings—and in whom other disorders have been excluded [3, 6].

Since the diagnosis of Silver-Russell syndrome is based primarily on identifying clinical features, studies assessing the oral changes present in this group of patients are important. According to reports in the literature, patients with SRS are predisposed to narrowing of the dental arches, crowding and class II occlusion (which occurs when the lower dental arch is posterior—more towards the back of the mouth than the upper one). Micrognathia is found in about 50% of subjects [7]. Abnormal shape and size of teeth, gothic palate and hypodontia involving premolar teeth have also been described in patients with Silver-Russell syndrome [9].

Patients with SRS require multidisciplinary care because they have a wide variety of health problems, which include growth disorders, severe feeding difficulties in early childhood, gastrointestinal problems, hypoglycemia, pubertal disorders, delayed motor and speech development, sleep apnea and psychosocial difficulties [6, 10]. Children should also receive dental and orthodontic care. It is important to disseminate knowledge about the dental problems of patients with this syndrome and their treatment needs.

The aim of the study was to evaluate phenotype features and oral health status in patients with Silver-Russell syndrome.

## Methods

Patients with Silver-Russell syndrome between the ages of 3 and 18, under the care of the Department of Genetics at the Children’s Memorial Health Institute, the Department of Orthodontics and the Department of Pediatric Dentistry at the Medical University of Warsaw, were eligible for the study. The control group consisted of generally healthy children of the same age reporting to the Department of Pediatric Dentistry, Medical University of Warsaw. The condition for inclusion in the study group was a diagnosis of Silver-Russell syndrome by a clinical geneticist. The exclusion criteria from the control group were other chronic general diseases or taking permanent medications (that may affect the oral cavity, e.g., anti-asthmatic, immunosuppressants); lack of written consent from the patient and/or parent/legal guardian, poor patient cooperation avoiding a complete examination. Written informed consent was obtained from study participants/parents before the study. The project was approved by the Medical University of Warsaw Bioethics Committee (No. KB/228/2009) and performed in compliance with the Declaration of Helsinki.

The study included analysis of medical records and clinical dental examination (intra- and extraoral). The extraoral examination focused on the shape and symmetry of the face, as well as the condition of the skin, lips, and corners of the mouth. Clinical examination was performed by one person to avoid bias. In the intraoral examination, the following were evaluated:

- Mucosa (presence, type, location of lesions), amount and consistency of saliva;
- Gingiva around all teeth (presence of malformations, recessions, gingivitis using the Gingival Index (GI) according to Löe [11]). The GI uses the following scoring system: 0 = normal gingiva; 1 = mild inflammation: a slight change in color, slight edema, no bleeding on probing; 2 = moderate inflammation: redness, edema, and glazing, or bleeding on probing; 3 = severe inflammation: marked redness and edema, the tendency toward spontaneous bleeding. It is assessed in all teeth in the oral cavity using 4 points (labial/buccal; lingual/palatal; mesial; distal).
- Tongue (mobility, tongue frenulum attachment, size);
- Palate (shape, height of articulation, developmental abnormalities);
- Occlusal conditions and the presence of teeth crowding, abnormal position of teeth in the arch;
- Dentition, including:
- The presence of teeth with carious cavities (code ≥ 3 according to ICDAS II [12]), fillings and missing teeth due to caries; caries frequency and intensity (DMFT) were determined [13];
- The presence of developmental abnormalities regarding the number of teeth, anatomical structure (shape, size) and enamel defects. In the case of suspected missing tooth buds, a pantomographic photograph was taken. A modified Developmental Enamel Defects Index (DDE index) was used to assess enamel developmental abnormalities [14].
- Oral hygiene status (Plaque Index, PLI) [11]. This index measures the plaque thickness on the gingival one-third of the teeth. It can be used on all teeth or selected (16, 12, 24, 36, 32, 44). Score criteria: 0—no plaque; 1—a film of plaque adhering to the free gingival margin and adjacent area of the tooth, which cannot be seen with the naked eye but only by using disclosing solution or a probe; 2—moderate accumulation of deposits within the gingival pocket, on the gingival margin and/ or adjacent tooth surface, which can be seen with the naked eye; 3—abundance of soft matter within the gingival pocket and/or on the tooth and gingival margin.

The results were statistically analyzed using Statistica 10 software. Comparisons of fractions (zero–one variables) between the two groups were made using the chi-square test, while comparisons of quantitative variables were made using the Mann–Whitney U test. A significance level of *p* < 0.05 was adopted.

## Results

Twenty-four patients with Silver-Russell syndrome (SRS) and 24 generally healthy children aged 3.25 to 17.9 years (mean age 8.56 ± 4.49) were examined. In each group, 10 children had deciduous dentition, 8 had mixed dentition, 6 had permanent dentition (Table 1). A pantomographic X-ray was performed in 10 patients with SRS.

**Table 1:** Characteristics of patients in the study group (Silver-Russell syndrome) and the control group

Group	Number of patients n/%	Gender		Mean age ± SD years)	Dentition		
		F/%	M/%		primary n/%	mixed n/%	permanent n/%
Silver-Russell Syndrome	24/100.00	12/50.00	12/50.00	9.04 ± 4.80	10/41.66	8/33.33	6/25.00
Controls	24/100.00	11/45.80	14/54.20	8.08 ± 4.18	10/41.66	8/33.33	6/25.00

In the study group, Silver Russel Syndrome was diagnosed only based on typical phenotypic features in 5 patients (normal karyotype). No other genetic investigations were performed on these patients. The diagnosis of these patients was made based on the Netchine-Harbison Clinical Scoring System (NH-CSS) when the children were under 2 years of age. There are six features:

- Small for gestational age (birth weight and/or length ≤ 2 SD for gestational age);
- Postnatal growth failure (length/height ≥ 2 SD below the mean at 24 months);
- Relative macrocephaly at birth (head circumference > 1.5 SD above birth weight and/or length);
- Frontal bossing or prominent forehead (Forehead projecting beyond the facial plane on a side view as a toddler [1–3 years]);
- Body asymmetry (limb length discrepancy ≥ 0.5 cm, or < 0.5 cm with ≥ 2 other asymmetric body parts);
- Feeding difficulties or BMI ≤ 2 SD at 24 months or current use of a feeding tube or cyproheptadine for appetite stimulation;

Clinical diagnosis is made when at least four of the clinical criteria are met, two of which must be relative macrocephaly at birth and frontal bossing.

Genetic confirmation of the diagnosis was obtained in 19 cases (maternal uniparental disomy of chromosome 7 (UPD7)—5 subjects; hypomethylation in the p15 region on the paternal copy of chromosome 11—14 subjects.

In the extraoral examination, patients with SRS were found to have a triangular facial shape (n = 22, 91.66% of those with the syndrome), facial asymmetry (n = 9; 37.5%), low-set, protruding ears (n = 19; 71.16%), narrow lips and downward-facing mouth angles (n = 21; 87.5% and n = 20; 83.33%, respectively). These features were not observed in children in the control group.

Hypotonia of the orbicularis oris muscle was noted in 7(29.17%) patients with Silver-Russell syndrome and 1(4.17%) child from the control group.

**Fig. 1:**
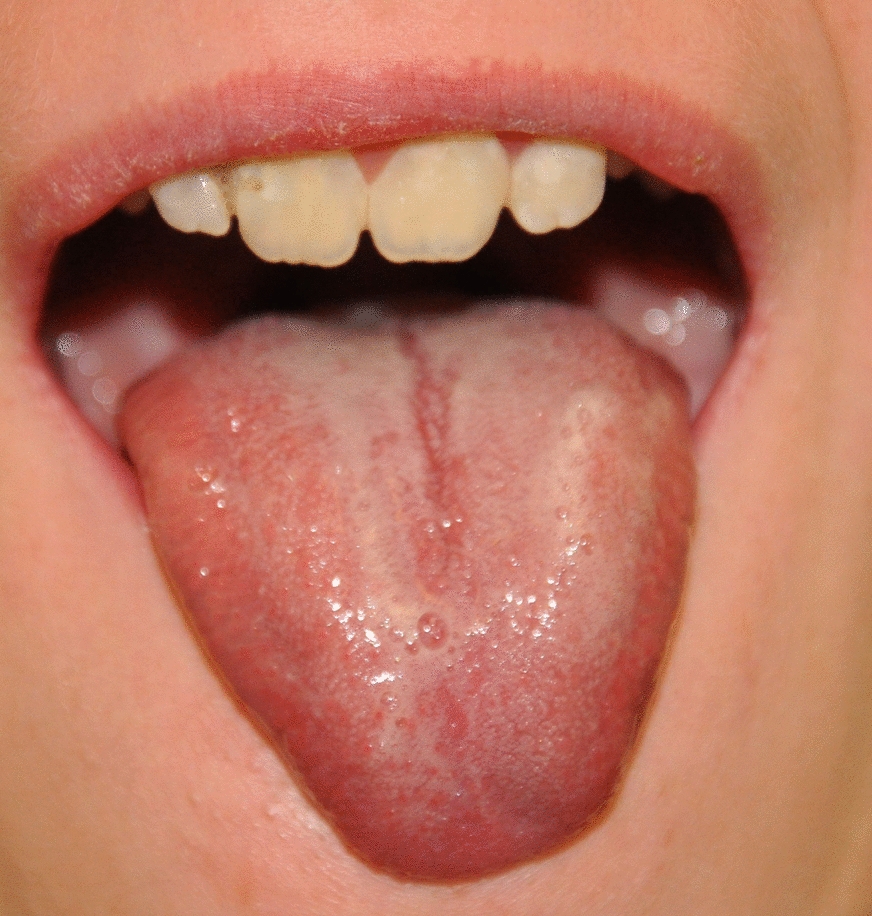
Narrow, dry lips, small tongue and frothy saliva in a boy aged 10 years with Silver-Russell syndrome

In intraoral examination, none of the subjects had lesions on the oral mucosa. Only subjects with SRS had reduced tongue dimensions (n = 7; 29.16%) (Fig. 1) and cleft palate (n = 1; 4.16%). A gothic palate was present in 19 (79.16%) subjects with SRS and 4 (16.66%) in the control group (*p* < 0.001).

**Fig. 2:**
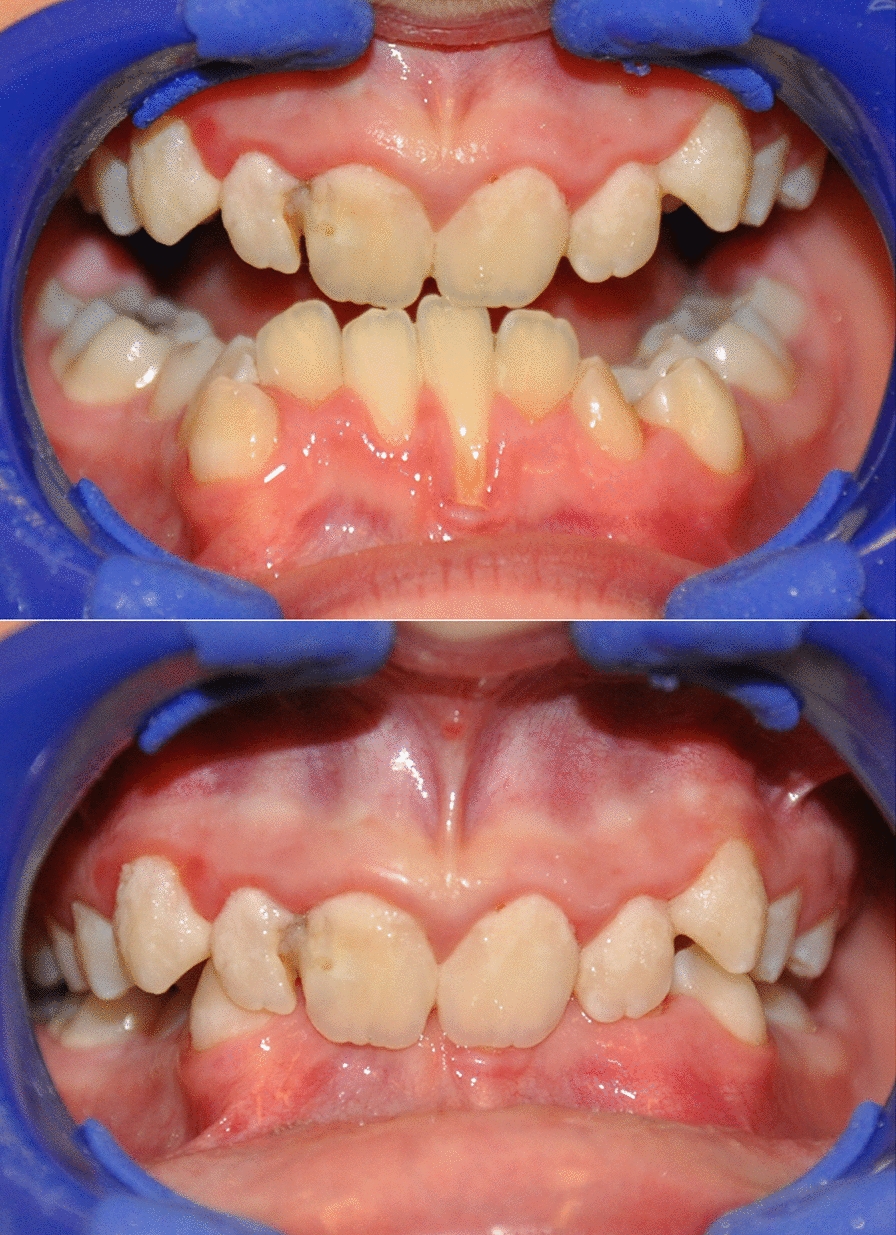
Hygiene and gingival status, dentition and occlusal conditions in a boy aged 10 years with Silver-Russell syndrome. Extensive plaque deposits, gingivitis, carious cavities, and bite abnormalities, such as crowding of teeth and deep bite, can be observed

There were no statistically significant differences in PLI values between the groups. Still, unsatisfactory hygiene was found more often in the SRS group than in the control group (16.66% vs. 4.16%, respectively (Table 2). GI values and the percentage of patients with gingivitis were statistically significantly higher in the Silver-Russell syndrome (p = 0.029). Six children in the SRS group had gingival recessions accompanied by moderate gingivitis, as well as crowding of teeth and occlusal abnormalities (class II occlusion with deep bite and class III with crossbite) (Fig. 2). The prevalence of caries was also higher in the group of subjects with Silver-Russell syndrome (p = 0.008). Six subjects in the SRS group (25%) had a clinically apparent reduction in saliva, which had a foamy or stringy consistency (Fig. 1). Oral health data for the study and control groups are summarized in Table 2.

**Table 2:** Oral health status of patients in the study and control groups

Parameter			SRS		Controls	p
Oral hygiene	PLI	mean ± SD	1.26 ± 0.86		1.02 ± 0.75	0.522
Gingiva	GI		0.58 ± 0.80		0.16 ± 0.36	0.029*
	GI ≥ 0,1	n/%	14/58.33		5/20.00	0.013*
	Gingival recessions		6/25.00		0/0.00	0.009*
Caries	Frequency		19/79.16		10/41.66	0.008*
	DMFT	mean ± SD	3.37 ± 5.01		2.00 ± 3.37	0.332
	DT		3.57 ± 4.32		2.06 ± 2.6	0.084
	MT		0.35 ± 1.33		0.20 ± 0.56	0.184
	FT		1.85 ± 1.87		1.20 ± 1.65	0.542
	dmft		3.38 ± 3.7		3.29 ± 3.68	0.910
	dt		3.44 ± 3.98		1.16 ± 1.82	0.014*
	mt		0.00 ± 0.00		0.05 ± 0.23	1.000
	ft		1.05 ± 1.73		3.16 ± 3.31	0.007*
Teeth number disorders	Hypodontia	n/%	2/8.0		0/0.00	0.149
	Retained teeth		3/12,5		0/0.00	0.074
Enamel defects			13 /54.16		2/8.33	< 0.001*
Anatomical disorders	Narrowing of the incisal edge		8/33.33		0	0.002*
	Vertical fissure on the labial surface of the incisal teeth		3/12.5		0	0.074
	Taurodontism		3/12.5	0		0.074
Malocclusion	Overall		17/70.83		10/41.66	0.042*
	Class II		11/45.83		10/41.66	0.771
	Crossbite		1/4.17		2/8.33	0.551
	Deep bite		12/50		0	< 0.001*
	Class III		1/4.17		0	0.312
Teeth crowding			18/75.0		8/33.3	0.004*

^*^p < 0.05

LEGENDS: SRS (Silver-Russell Syndrome); DMFT/dmft (decayed,missing,filled teeth in permanent or primary dentition respectively); DT/dt (decayed permanent/primary teeth); MT/mt (missing permanent/primary teeth); FT/ft (filled permanent/primary teeth)

In subjects with Silver-Russell syndrome, enamel malformations were observed more often than in the control group (*p* < 0.001). In generally healthy children, these were exclusively enamel opacities of permanent teeth. In the study group, they were observed in both deciduous (5 subjects) and permanent teeth (8 subjects). Of the 18 children with deciduous teeth, 2 had opacities, 2 had enamel hypoplasia, and 1 had a combination of defects. Among the 14 subjects with permanent teeth, 4 children had opacities, 2 had hypoplasia, and 2 had a combination of defects (Table 2).

**Fig. 3:**
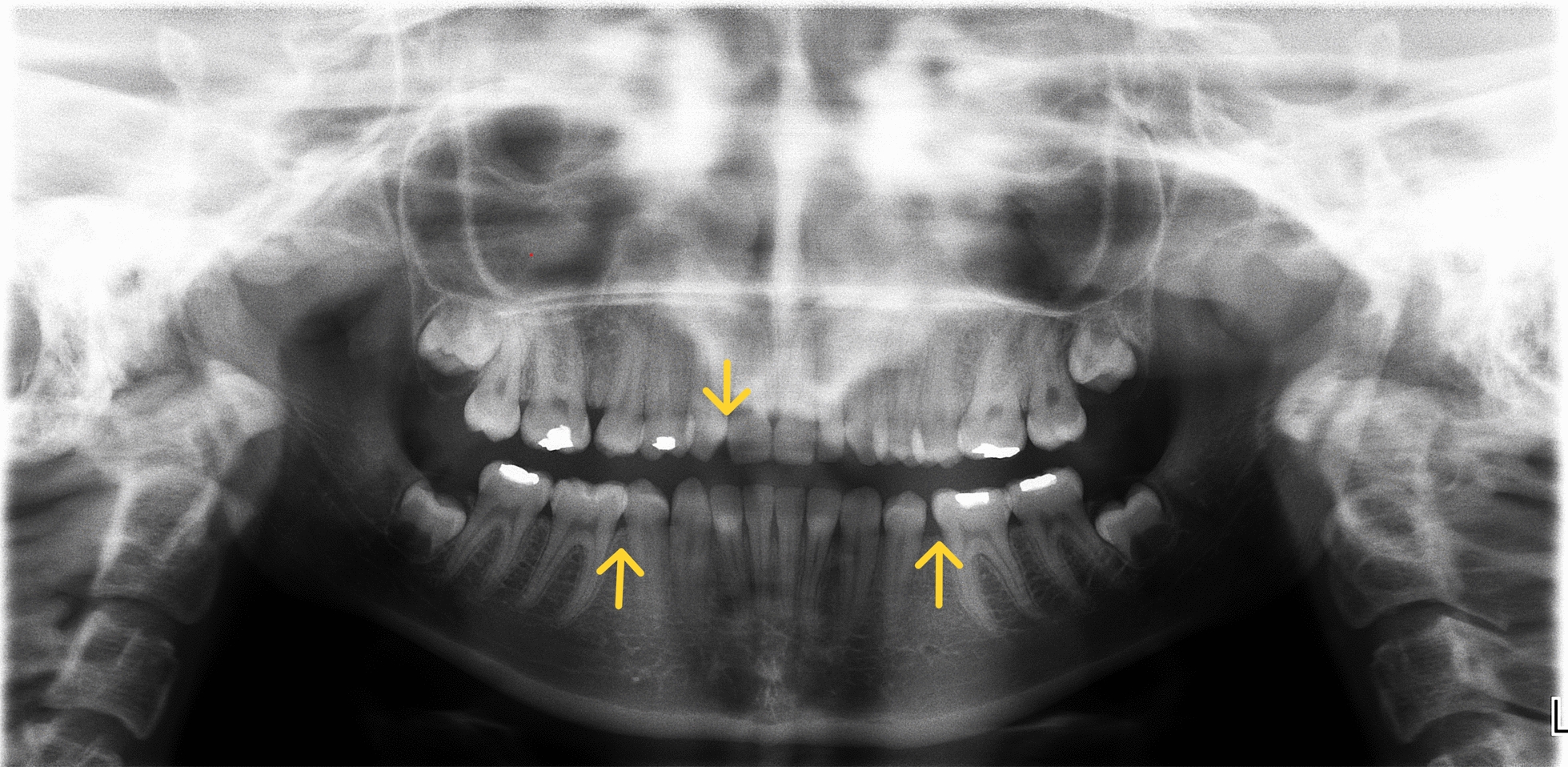
Hypodontia in a girl with SRS: missing teeth 12 (upper right lateral incisor), 35, 45 (lower right and left second premolars)

**Fig. 4:**
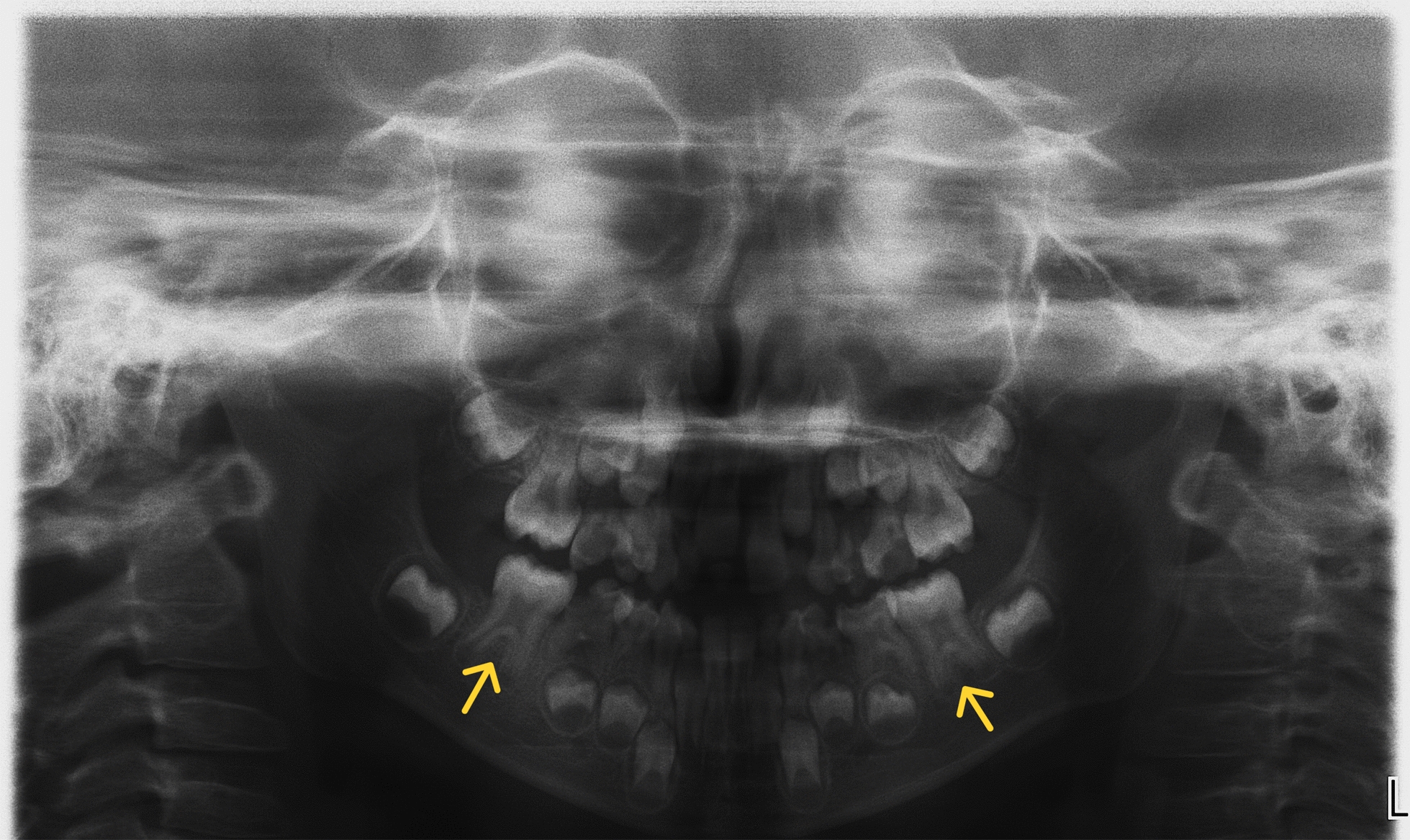
Taurodontism (a developmental disorder involving an increase in the size of the tooth chamber and a shift of the furcation of the roots toward their apices) of first permanent molars in a 7-year-old female patient with SRS

Abnormalities regarding the number of permanent teeth and anatomical structure were reported only among patients with Silver-Russell syndrome (hypodontia—3 cases, retained teeth—3 cases, taurodontism—3 cases, crown structure abnormalities—11 cases) (Figs. 3 and 4).

In Silver-Russell syndrome, occlusal abnormalities were also more common than in the control group (P = 0.002). In the study group, these were mainly class II defects (45.83%), deep bites (50%) and crossbites (29.16%). In the control group, 41.66% of the children showed the presence of class II occlusion, which in two girls co-occurred with a crossbite. In the group of generally healthy children, there were no deep bites or class III occlusion. Teeth crowding was present among 75% of patients with Silver-Russell syndrome, compared to 33% in generally healthy patients (p = 0.004). (Table 2).

The group is too small to perform statistical analysis regarding the specific genotypes found in patients. However, Table 3 summarizes the features observed in the oral cavity, considering the frequency of their occurrence in a specific genotype (Table 3). This indicates that there may be a correlation between the UPD7 genotype and class III malocclusion or crossbite, but it should be confirmed in a larger study. Genotypes were determined for 19 of 24 SRS patients. The remaining patients showed no genetic variation compared to generally healthy patients, so they were not included in the Table.

**Table 3:** Oral and dental conditions in specific genotypes of patients with Silver-Russell Syndrome

Feature	UPD7 [n/%] Total N = 5/19	Hypomethylation [n/%] Total N = 14/19
Number of patients with specific disorder		
Enamel defects		
Total	2	11
Opacities	2	5
Hypoplasia	0	6
Caries		
Present	3	12
Absent	2	2
Teeth number disorders		
Hypodontia	0	2
Retained teeth	0	1
Malocclusion		
Class II	2	7
Crossbite	1	0
Deep bite	2	8
Class III	1	0
Teeth crowding	4	12
Gothic palate		
Present	5	12
Absent	0	2

## Discussion

In the available literature, there are few reports presenting the characteristic oral phenotypic features observed in patients with Silver-Russell syndrome. These are usually descriptions of small groups of patients or single case reports. Authors typically focus on analyzing extraoral features characteristic of the syndrome and orthodontic diagnosis [5, 7, 8, 10, 15–17]. Among the few publications on the oral health status of patients with Silver-Russell syndrome, there is little information on the presence of characteristic intraoral phenotypic features and the intensity of caries, hygiene and gingival status in these patients. Knowing these is important both in the diagnostic process and in planning dental care, Vo Quang et al. [8] conducted a study to define the maxillofacial phenotype in patients with SRS. The study included 31 patients with SRS. Considering the rarity of the disease, this is a large group of subjects. The spectrum of facial dysmorphism is often incomplete in patients with SRS. However, new characteristics were demonstrated: small forehead, small mandible, skeletal class II, crowding of teeth in the lower arch and overbite. These observations are consistent with the results obtained in the above study.

According to reports by most researchers, patients with Silver-Russell Syndrome have a predisposition to the presence of narrowing of dental arches, crowding and Class II defects [2, 3, 8, 15], gothic palate and hypodontia involving premolar teeth, as well as micrognathia and microdontia [7, 10, 15, 18, 19] and disorders of tooth shape and size [2, 5, 8]. In the above study, we noted with a high frequency the presence of a gothic palate among patients with SRS. Marczak-Hałupka et al. [19], in a review paper describing the characteristics of patients with SRS, point to the presence of a high palate and hypodontia of premolar teeth as one of the main oral manifestations, which was also observed in the above study. The incidence of gothic palate in SRS patients is also confirmed by the studies of Jagielska et al. [20] and Cammarata-Scalisi et al. [21]. According to Cammarata-Scalisi et al. [21], gothic palate occurs in patients with hypomethylation in the 11p15.5 region.

In the present study, in addition to Class II defects, we also noted a trend toward deep or crossbite in patients with Silver-Russell Syndrome. Defects of this type have also been noted by other researchers [5, 22]. Wakeling et al. [1] emphasize that the upper dental arch is often narrow and crowded, and with coexisting facial asymmetry, a crossbite can occur, making it difficult for patients to chew properly. Despite the tendency toward a skeletal open bite in children with SRS, Bergman et al. [9] showed that as many as 31% of patients exhibited a deep bite, indicating compensatory alveolar growth. Facial asymmetry was assessed by body asymmetry, but no facial asymmetry of clinical significance was found. The prevalence of malocclusion was also higher in children with SRS, which may lead to an increased need for orthodontic treatment. Interestingly, Class II malocclusions were observed with a similar frequency to those of the control group. The possible correlation of crossbites with SRS is caused by uniparental disomy of chromosome 7 (UPD7). The literature reports that patients with UPD7 may represent distinct phenotypic entities of SRS, defined as a benign phenotype [23].

Reports of the occurrence of cleft palate in SRS patients have also been encountered in the literature [2, 15, 17], but we did not observe this abnormality frequently among the patients we studied (1 case out of 24 patients examined). Patients with cleft palate have been described by Khalid et al. [15] and Shiraishi et al. [17]. Genetic analysis of the patients was performed, and H19-DMR hypomethylation was shown at chromosome 11, but no methylation was found at chromosome 7 in these patients [17]. Cleft palate or cleft uvula was found to occur in 7% of patients with ICR1 hypomethylation and in no patients with UPD7 [3]. This is supported by a study by Wakeling et al. [2], which found cleft palate or uvula only in patients with 11p15.5 hypomethylation.

A feature observed only in SRS patients in the above study was a reduction in tongue size, occurring in 29.16% of children. However, there are no reports in the literature on this subject.

Also, anatomical abnormalities in both permanent and deciduous teeth were found among children with SRS. Narrowing of the incisal edges and the presence of a vertical fissure on the labial surface of the tooth were noted. Abnormalities of tooth shape and size in patients with SRS were also described by Eggermann et al. [24], Ribeiro et al. [25] and Reddy et al. [26]. More than half of our SRS patients had enamel defects (54.16%). Opacities, hypoplasia and a combination of defects were observed. Hypoplasia and hypomineralization of tooth enamel were found in both the deciduous and permanent dentition in about 50% of the children studied by Kotilainen et al. [27]. They also observed that the morphology of the occlusal surface of some permanent first molars was atypical.

The occlusal and developmental abnormalities of the dentition (abnormal morphological structure, impaired formation of protein structure and mineralization) observed in the study group may promote bacterial biofilm retention and increase the risk of dental caries and gingivitis. Accumulation of plaque may also be facilitated by impaired oral self-cleaning mechanisms. Wakeling et al. [1] report that muscular hypotonia is typical, especially in younger children with SRS. Among almost 30% of our patients with Silver-Russell syndrome, we observed hypotonia of the orbicularis oris muscle and a reduction in the size of the tongue, as well as a low amount of resting saliva with a foamy or stringy consistency (25% of children).

Caries intensity was higher in the study group, both in deciduous and permanent teeth. The risk of tooth decay was also reported by other authors [7, 26]. Przystupa et al. [7] emphasize that the susceptibility to dental caries of SRS patients is not insignificant for the development of the stomatognathic system. Patients with anatomical features that predispose to the development of dental caries and accompanying hygienic negligence on the part of caregivers lead to tooth loss due to caries already in the first years of a child’s life. Gingivitis was observed twice as often in children with Silver-Russell syndrome as in the control group. There is a lack of information in the literature regarding changes in periodontal tissues in this syndrome. Certainly, however, predisposing factors of plaque accumulation and mouth breathing promote gingivitis, and malocclusion promotes gingival recession.

Unfortunately, in the above paper, we did not analyze potential oral health risks that may be associated with feeding disorders present in SRS patients (e.g., feeding difficulties or the need for enteral feeding). We can describe this as a kind of limitation of our study. Nevertheless, it should be mentioned that these disorders may be a factor in developing oral conditions in this group of patients. Common feeding problems for children with SRS were poor appetite, fussiness, slow feeding, and problems associated with oral-motor dysfunction [28]. This can promote the risk of giving children foods they are more willing to accept, such as sweet foods or those with a mushy, lagging consistency. This is a factor in the development of caries. Avoidance of chewing also leads to weak development of dental arches, which can predispose to malocclusion. Another aspect is the frequent occurrence of gastroesophageal reflux. Chronic regurgitation of gastric acids in patients with gastroesophageal reflux may cause dental erosion.

## Conclusions

Administering dental care to individuals with a genetic condition is challenging for the clinician. It requires knowledge of the oral anomalies of these syndromes and also of the risk factors for oral diseases associated with their occurrence. Prompt orthodontic and dental intervention in children with SRS can help normalize oral function and facial appearance.

## Data Availability

The data that support the findings of this study are not openly available due to sensitivity reasons and are available from the corresponding author upon reasonable request. Data are located in controlled access data storage at the Medical University of Warsaw. The datasets used and/or analyzed during the current study are available from the corresponding author upon reasonable request.
